# Supplementary material for: Genetic Diversity of the Human Adenovirus C Isolated from Hospitalized Children in Russia (2019–2022)
Source: Viruses. 2024 Feb 29;16(3):386. doi: 10.3390/v16030386 (PMC10975726; doi:10.3390/v16030386)
Supplement: Supplementary file 1 [file viruses-16-00386-s001.zip › viruses-2801409-supplementary.pdf]

Supplementary. Table S1. Pairwise identities of human adenovirus C nucleotide sequences from the study on strains of genotypes C1, C2, C5, C6, C57, C89, and C89.

		P1H1F1	P2H2F2	P5H5F5	P6H6F6	P1H5F6	P89H2F2	P1H2F2
		C1	C2	C5	C6	C57	C89	C108
	Ref Acc #	JX173086	MK836309	OR753108	JX423389	HQ003817	MH121097	OQ108498
HAdVC/Novosibirsk/8.65Hp/2020	Complete genome	**99,583**	95,407	95,183	95,586	95,778	95,394	95,801
	P	99,594	99,652	99,183	99,536	**99,710**	98,365	**99,710**
	H	**99,793**	86,674	84,907	85,321	88,741	86,409	87,022
	F	**99,485**	72,884	75,502	77,631	77,694	72,884	72,827
HAdVC/Novosibirsk/8.135Hl/2021	Complete genome	**99,480**	95,289	95,077	95,639	95,805	95,298	95,669
	P	99,594	99,652	99,299	99,652	**99,710**	98,599	**99,710**
	H	**99,793**	86,674	84,837	85,356	88,776	86,409	87,022
	F	**99,200**	72,942	75,273	77,379	77,190	72,942	72,884
HAdVC/Novosibirsk/8.81V/2020	Complete genome	**99,497**	95,301	95,093	95,647	95,819	95,312	95,688
	P	99,652	99,710	99,358	99,710	**99,768**	98,541	**99,768**
	H	**99,793**	86,674	84,837	85,356	88,776	86,409	87,022
	F	**99,200**	72,942	75,273	77,379	77,190	72,942	72,884
HAdVC/Novosibirsk/8.142V/2020	Complete genome	95,495	**99,493**	95,113	97,220	96,919	99,124	99,048
	P	99,184	99,242	98,774	99,242	**99,301**	98,592	**99,301**
	H	86,708	**99,862**	84,236	90,108	89,709	97,730	98,693
	F	73,230	**99,657**	73,230	75,047	74,984	99,543	99,600
HAdVC/Novosibirsk/7.45Hl/2021	Complete genome	95,524	**99,527**	95,142	97,244	96,948	99,157	99,084
	P	99,184	99,242	98,774	99,242	**99,301**	98,592	**99,301**
	H	86,778	**99,966**	84,342	90,143	89,744	97,833	98,796
	F	73,230	**99,657**	73,230	75,047	74,984	99,543	99,600
HAdVC/Novosibirsk/7.17Hl/2021	Complete genome	95,509	**99,516**	95,136	97,234	96,933	99,143	99,073
	P	99,184	99,242	98,774	99,242	**99,301**	98,592	**99,301**

	H	86,778	**99,966**	84,342	90,143	89,744	97,833	98,796
	F	73,172	**99,600**	73,287	74,984	74,921	99,485	99,543
	Complete genome	95,520	**99,521**	95,136	97,245	96,941	99,149	99,076
	P	99,242	99,301	98,832	99,301	**99,359**	98,650	**99,359**
	H	86,778	**99,966**	84,342	90,143	89,744	97,833	98,796
HAdVC/Novosibirsk/8.202V/2020	F	73,230	**99,657**	73,230	75,047	74,984	99,543	99,600
	Complete genome	95,546	**99,424**	95,147	97,186	97,011	99,034	99,120
	P	99,301	99,359	98,891	99,359	**99,417**	98,592	**99,417**
	H	86,778	**99,966**	84,342	90,143	89,744	97,833	98,796
HAdVC/Novosibirsk/7.134V/2019	F	73,230	**99,657**	73,230	75,047	74,984	99,543	99,600
	Complete genome	95,371	95,099	**99,473**	95,124	95,075	95,031	95,053
	P	99,241	99,299	**100,000**	99,416	99,358	98,589	99,358
	H	85,082	84,131	**97,726**	84,567	85,925	83,585	83,920
HAdVC/Novosibirsk/7.273Hp/2022	F	75,100	72,874	**99,370**	71,708	71,645	73,046	72,816
	Complete genome	95,294	95,086	**99,660**	95,116	95,036	94,988	94,995
	P	99,358	99,416	**99,766**	99,533	99,475	98,707	99,475
	H	84,907	84,377	**99,895**	84,602	86,031	83,831	84,096
HAdVC/Novosibirsk/7.2Hp/2022	F	75,043	72,874	**99,885**	71,582	71,393	73,046	72,816
	Complete genome	95,496	99,017	95,000	97,192	96,759	**99,606**	98,908
	P	98,307	98,365	98,304	98,599	98,424	**99,650**	98,424
	H	86,479	97,730	83,726	90,745	88,974	**99,863**	97,454
HAdVC/Novosibirsk/8.234V/2020	F	73,230	99,543	73,345	75,110	75,047	**99,886**	99,600
	Complete genome	95,776	99,128	95,071	97,209	96,917	99,073	**99,610**
	P	99,768	99,826	99,358	99,710	**99,884**	98,541	**99,884**
	H	86,987	98,830	84,096	90,178	89,639	97,592	**99,931**
HAdVC/Novosibirsk/8.171V/2020	F	73,172	99,657	73,230	75,110	75,047	99,657	**99,828**

Note (for Supplementary material - Table S1): the white color mean the lowest identity value in a row; the green color mean the highest identity value in a row.
